# Comparing language outcomes in monolingual and bilingual stroke patients

**DOI:** 10.1093/brain/awv020

**Published:** 2015-02-12

**Authors:** Thomas M. H. Hope, ‘Ōiwi Parker Jones, Alice Grogan, Jenny Crinion, Johanna Rae, Louise Ruffle, Alex P. Leff, Mohamed L. Seghier, Cathy J. Price, David W. Green

**Affiliations:** 1 Wellcome Trust Centre for Neuroimaging, University College London, UK; 2 Wolfson College, University of Oxford, UK; 3 Institute of Cognitive Neuroscience, University College London, UK; 4 Department of Brain, Repair and Rehabilitation, Institute of Neurology, University College London, UK; 5 Experimental Psychology, University College London, London WC1E 6BT, UK

**Keywords:** bilingualism, stroke, language, aphasia, prognosis

## Abstract

Post-stroke prognoses are usually inductive, generalizing trends learned from one group of patients, whose outcomes are known, to make predictions for new patients. Research into the recovery of language function is almost exclusively focused on monolingual stroke patients, but bilingualism is the norm in many parts of the world. If bilingual language recruits qualitatively different networks in the brain, prognostic models developed for monolinguals might not generalize well to bilingual stroke patients. Here, we sought to establish how applicable post-stroke prognostic models, trained with monolingual patient data, are to bilingual stroke patients who had been ordinarily resident in the UK for many years. We used an algorithm to extract binary lesion images for each stroke patient, and assessed their language with a standard tool. We used feature selection and cross-validation to find ‘good’ prognostic models for each of 22 different language skills, using monolingual data only (174 patients; 112 males and 62 females; age at stroke: mean = 53.0 years, standard deviation = 12.2 years, range = 17.2–80.1 years; time post-stroke: mean = 55.6 months, standard deviation = 62.6 months, range = 3.1–431.9 months), then made predictions for both monolinguals and bilinguals (33 patients; 18 males and 15 females; age at stroke: mean = 49.0 years, standard deviation = 13.2 years, range = 23.1–77.0 years; time post-stroke: mean = 49.2 months, standard deviation = 55.8 months, range = 3.9–219.9 months) separately, after training with monolingual data only. We measured group differences by comparing prediction error distributions, and used a Bayesian test to search for group differences in terms of lesion-deficit associations in the brain. Our models distinguish better outcomes from worse outcomes equally well within each group, but tended to be over-optimistic when predicting bilingual language outcomes: our bilingual patients tended to have poorer language skills than expected, based on trends learned from monolingual data alone, and this was significant (*P* < 0.05, corrected for multiple comparisons) in 13/22 language tasks. Both patient groups appeared to be sensitive to damage in the same sets of regions, though the bilinguals were more sensitive than the monolinguals.

## Abstract (precis)

Hope *et al.* compare language outcomes in monolingual and bilingual stroke patients, and find that prognostic models based on monolingual data alone overestimate language skills in bilingual patients. Both groups seem sensitive to damage in the same brain regions, but bilinguals appear more sensitive to that damage than monolinguals.

## Introduction

Research suggests that language deficits, or aphasia, are some of the most feared consequences after stroke (Lam and Wodchis, 2010). Patients with aphasia suffer disproportionate levels of anxiety, depression and unemployment, at just the same time as their most basic coping mechanism—talking with family and friends—is being undermined. Stroke patients want to know whether, when, and in what respects they might hope to recover lost language skills (Hanger *et al.*, 1998; Choi-Kwon *et al.*, 2005)—questions that have motivated a great deal of research into the factors that predict better or worse recovery from post-stroke aphasia (Keenan and Brassell, 1974; Miceli *et al.*, 1981; Pickersgill and Lincoln, 1983; Basso, 1992; Pedersen *et al.*, 1995; Maas *et al.*, 2012; Plowman *et al.*, 2012; El Hachioui *et al.*, 2013). This work has traditionally emphasized monolingual patients, with bilingualism treated as a special case of language use which, though relevant and interesting, might not be taken to provide the kind of primary evidence that the study of post-stroke aphasia requires. But bilingualism (hereafter used to indicate anyone who speaks more than one language) is the norm rather than the exception in many parts of the world; here, we explore if and how prognostic models built from monolingual stroke patient data can be generalized to bilingual patients.

### Language in the bilingual brain

Research suggests that bilingualism induces plastic changes in the brain (Mechelli *et al.*, 2004; Bialystok *et al.*, 2009), but the functional significance of these changes is still debated. On the one hand, a non-native language may be represented and processed differently from that of the native language. For example, where one language is learned later than another, neural regions may become specialized for the representation of the native language and resist recruitment by a non-native language, and there are also claims that some (e.g. temporo-parietal) regions may preferentially process second languages (Lucas *et al.*, 2004). Research using electrical stimulation in bilingual patients suffering from seizures (Lucas *et al.*, 2004; Cervenka *et al.*, 2011) or with left-frontal glioma (Bello *et al.*, 2006), indicates sites where the stimulation disrupts object naming in a language-specific manner (together with other sites where the effect is common to both). These results encourage the view that there is neural divergence in the underlying language processes. But object naming involves a network of regions, mapping lexical representations into speech (Price, 2012) and as suggestive as these data may be, they are still consistent with the notion that first and subsequent languages use a common network, which is differentially sensitive to disruption as a function of the language in use.

A more specific model of bilingual language processing, the ‘procedural/declarative’ model (Ullman, 2001; Paradis, 2004), contends that native and non-native languages, their grammatical aspects in particular, are served by distinct neural circuits. The distinction flows from the intuition that native language grammar is learned in an implicit or procedural manner, associated with left frontal (i.e. anterior) regions and the basal ganglia, while second and subsequent language grammars are learned more explicitly, with a greater emphasis on declarative memory in temporo-parietal (i.e. posterior) regions (Ullman, 2001). This model naturally predicts different activation profiles in functional imaging studies when bilingual speakers process spoken sentences in their native and non-native languages (Dehaene *et al.*, 1997; Yokoyama *et al.*, 2006), and is also supported by neuropsychological case reports associating selectively impaired recovery in the native language with damage to basal ganglia structures (Fabbro and Paradis, 1995; Garcia-Caballero *et al.*, 2007), and selectively preserved grammatical processing in the native language with the preservation of the same structures (Zanini *et al.*, 2011). However, other neuropsychological studies point to grammatical deficits in both first and subsequent languages arising from anterior lesions, contrary to the procedural/declarative model’s predictions (Tschirren *et al.*, 2011).

An alternative account of bilingual language processing—the neural convergence account—argues that identical regions mediate monolingual and bilingual language (Green, 2008; Consonni *et al.*, 2013) both during lexical processing (Parker Jones *et al.*, 2012) and grammatical processing (Abutalebi, 2008). Although common regions are active for native and non-native languages, the functional demand on these regions is higher for bilingual speakers (Abutalebi and Green, 2007; Parker Jones *et al.*, 2012). Such increased demand arises from the reduced proficiency of language processing in bilingual speakers for their two languages compared to that of monolingual speakers of those languages (Portocarrero *et al.*, 2007; Bialystok *et al.*, 2009) and from the concurrent activation of both languages whilst processing just one language (Kroll *et al.*, 2006; Hoshino *et al.*, 2011). Under the neural convergence account, the apparent differences between how first and subsequent languages are processed reflect an increased reliance on cognitive control mechanisms to mediate between the different languages, rather than differences in their neural representation. Selective recovery of language post-stroke would also be attributable to difficulties in exercising language control (Abutalebi and Green, 2007; Green *et al.*, 2010). 

If the neural convergence account of bilingual language is correct, language deficits and recovery in bilingual stroke patients should reflect damage in essentially the same brain regions as monolingual stroke patients, although the sensitivity to damage may be greater in bilinguals, either because of reduced premorbid language proficiency, or because bilinguals are doing more with the same regions than monolinguals. By contrast, if there is distinct representation, or neural divergence, the ‘critical regions’ for each patient group might be very different. The practical implications are profound because: (i) lesion site and size information are increasingly recognized as key prognostic factors in post-stroke aphasia (Plowman *et al.*, 2012; Hope *et al.*, 2013); (ii) the best predictions can only be made by excluding irrelevant regions (Hope *et al.*, 2013); and (iii) most practical attempts to predict prognoses for stroke patients are inductive, in the sense that they aim to learn (often statistical) trends from patients whose outcomes are known, and to generalize those trends to new patients (Jorgensen *et al.*, 1995; Tilling *et al.*, 2001; Pettersen *et al.*, 2002; Price *et al.*, 2010; Toschke *et al.*, 2010; Zhu *et al.*, 2010; Hope *et al.*, 2013). If the neural convergence theory is right, the search for critical language regions in monolingual stroke patients should be equally applicable to bilingual patients: both patient groups can be pooled, with work in each group yielding lessons that are relevant to all. But if there are divergent representations, lesion-deficit associations learned from the monolingual stroke patients may not tell us much about bilingual stroke patients, which in turn implies that we need to repeat the whole model-selection process for monolingual and bilingual stroke patients separately.

In what follows, we ask how applicable prognostic models designed for monolingual English stroke patients are to language recovery in bilingual stroke patients (non-native speakers of English). Specifically, we ask (i) whether the bilingual patients’ English language outcomes are better, worse, or similar to expectations based on monolingual data; and (ii) whether the apparent differences that we do in fact find correspond to differences at the level of lesion-deficit associations in the brain. Neural divergence accounts of bilingual language predict that that there should be significant lesion-deficit associations for the bilingual group in regions where there is no association at all for the monolingual patients (and the procedural/declarative model predicts that those differences will principally be found in regions implicated by syntactic language processing). By contrast, common lesion-deficit associations are predicted by the neural convergence account albeit with the possibility of significant differences within regions that play the same roles in both groups (i.e. suggesting that the load on those regions may differ across groups).

## Materials and methods

### The PLORAS database

Our patient data are extracted from our *PLORAS* database (Price *et al.*, 2010), which associates stroke patients, tested over a broad range of times post-stroke, with demographic data, behavioural test scores from the Comprehensive Aphasia Test (Swinburn, 2004), and high resolution T_1_-weighted MRI brain scans.

### Patient selection

We included all available right-handed patients with left hemisphere stroke irrespective of the presence or absence of aphasia or any other type of cognitive impairments (e.g. spatial neglect). Patients were only excluded on the basis of their behaviour if they were unable to consent for the study, had contraindications to MRI scanning or were unable to see or hear the stimuli required to assess their language abilities. Additionally, patients were excluded if they: (i) were <3 months post-stroke when assessed; (ii) had evidence of other significant neurological conditions (e.g. dementia, multiple sclerosis); (iii) showed no visible damage anywhere in the brain, as assessed by a neurologist (A.P.L.), using the patients’ raw T_1_-weighted scans; or (iv) had suffered dispersed rather than focal damage. To make this last judgement, we employed the lesion identification algorithm described previously (Seghier *et al.*, 2008) to identify the damaged regions in the patients’ brains, and excluded patients whose lesions occupied a contiguous volume of <1 cm^3^ in the left hemisphere of the brain—reflecting the spatial scale at which the patients’ scans are smoothed when compared to control data. This selection process yielded 174 stroke patients who are native English speakers (the monolingual group), and 33 stroke patients who are non-native English speakers (the bilingual group).

The monolingual group included 112 males and 62 females, and the bilingual group included 18 males and 15 females. Age at onset was variable within each group [monolinguals’ mean = 53.0 years; standard deviation (SD) = 12.2 years: bilinguals’ mean = 49.0 years; SD = 13.2 years], but not significantly different across groups (t = 1.68, *P* = 0.10). And the same was true for time since stroke at assessment (monolinguals’ mean = 55.6 months; SD = 62.6 months; bilinguals’ mean = 49.2 months; SD = 55.8 months; between group comparison: t = 0.54, *P* = 0.59). We also compared the number of years of education after the age of 16 across groups (with missing data for 5/174 monolinguals and 2/33 bilinguals); the monolingual patients reported slightly more years on average than bilinguals (1.75 years versus 1.32 years), but the difference was not significant (Wilcoxon rank sum test: z = 1.03, *P* = 0.30).

### Bilinguals’ language histories and experience of English

All of the patients in our bilingual group were ordinarily resident in the UK, both at the time of testing and before their strokes occurred. They had a large variety of first languages, including: Dutch, Farsi, Finnish, French (four patients), German (two patients), Gujerati (three patients), Hakka (two patients), Ibo, Italian (three patients), Jamaican Patois, Lithuanian, Lunda, Mauritian, Polish, Portugese, Punjabi (two patients), Russian, Serbian, Spanish, Swahili, Tamil (two patients), and Yoruba. We collected several other types of language history data (Table 1), though every patient left at least some of our questions here unanswered. Most of the bilingual patients spoke more than two languages fluently, and although 11 reported that they were bilingual from birth, none reported learning English before they were 2 years old. Twenty per cent of the patients who gave us the information reported using English less than half the time pre-stroke, rising to 30% post-stroke. Exactly half of those who answered gave themselves the highest rating (9/9) for pre-stroke proficiency in English, and three (11%) gave themselves a rating below 7/9.

**Table 1:** Language history and immersion data

Language use data	*n*	Mean	Maximum	Minimum
Number of languages spoken	30	3.3	8	2
Age of bilingualism (years)	26	5.5	21	0
Age learned English (years)	24	10.7	24	2
Years English used	24	39.8	57	9
Self-rated premorbid proficiency (1 = lowest; 9 = highest)	26	8.1	9	5.8
Self-rated % time spent using English pre-stroke	15	73.6	100	33.3
Self-rated % time spent using English post-stroke	27	64.9	100	15

*n* = number of patients who responded to each question; Maximum = the maximum value reported by any patient; Minimum = the minimum value reported by any patient.

### Behavioural data

Each patient was assigned a behaviour score based on the tasks tested in the Comprehensive Aphasia Test. For ease of comparison across tasks, these raw scores are then converted to T-scores, representing each patient’s assessed skill on each task (e.g. describing a picture; reading non-words) relative to a reference population of 60 aphasic patients. The threshold for impairment is defined relative to a second population of 27 neurologically normal controls such that performance below threshold would place the patient in the bottom 5% of the normal population. Lower scores indicate poorer performance. We excluded six scores relating to certain non-linguistic cognitive skills (line bisection, semantic memory, recognition memory, gesturing object use, arithmetic and memory). The groups did not differ in any of these scores (all t < 1.0 or *P* > 0.10). We also excluded six additional scores that merely summarize other scores. Our analyses therefore concern the remaining 22 language scores from the Comprehensive Aphasia Test (see Table 2 for the task scores).

**Table 2:** Behaviour scores for the two patient groups

Task	Monolingual patients						Bilingual patients					
	Sample		Scores				Sample		Scores			
	*n*	Impaired	Min	Max	Mean	SD	*n*	Impaired	Min	Max	Mean	SD
Fluency	174	58	37	75	61.4	9.9	32	18	37	70	56.3	8.3
Comprehension (spoken words)	174	85	28	72	59.0	8.5	31	23	37	72	54.5	8.4
Comprehension (spoken sentences)	171	27	34	60	54.3	7.7	30	12	34	60	49.4	9.2
Comprehension (spoken para.)	174	49	32	65	57.7	7.0	32	18	41	65	53.5	7.7
Comprehension (written words)	172	56	28	72	60.5	8.4	31	21	43	67	54.0	6.8
Comprehension (written sentences)	172	69	36	73	60.4	8.2	31	25	41	68	53.9	6.9
Repeating words	173	73	35	65	57.2	8.5	31	17	35	65	53.8	9.2
Repeating complex words	173	61	38	62	56.1	8.9	31	15	38	62	52.5	10.8
Repeating non-words	173	49	38	67	56.5	9.1	32	14	38	67	53.1	9.2
Repeating digits	173	60	35	66	55.2	8.9	33	14	35	66	52.8	9.2
Repeating sentences	173	74	39	63	56.4	8.7	32	21	39	63	52.3	9.5
Object naming	174	86	37	74	61.6	10.2	32	24	37	74	55.1	9.3
Action naming	174	97	39	69	58.8	9.2	32	26	39	69	51.5	10.2
Spoken picture description	171	93	39	75	58.9	8.8	33	28	39	67	53.7	6.5
Reading words	172	94	38	69	58.9	9.3	32	23	38	69	54.5	8.8
Reading complex words	171	77	40	67	57.7	10.7	32	20	40	67	55.2	10.7
Reading function words	171	24	35	62	57.6	8.6	32	5	35	62	56.6	8.3
Reading non-words	171	77	40	68	57.3	10.8	32	19	40	68	54.3	10.9
Writing (copying)	170	14	40	61	59.3	4.6	28	7	40	61	56.4	6.6
Writing (picture naming)	173	33	38	67	60.5	8.0	31	15	38	67	55.4	8.3
Writing (dictation)	173	66	38	68	59.2	8.6	30	21	38	68	53.9	6.7
Written picture description	167	78	42	75	64.1	8.5	31	24	42	71	56.9	9.3

Including the minimum (Min), maximum (Max), mean and standard deviations for each language score by group, together with the number of patients in each group who might be considered ‘impaired’, in the sense that their performance on that task fell within the lower 5% of scores relative to a reference population of neurologically normal controls. For ease of comparison, all scores are converted into T-scores, using the procedure described in Swinburn *et al.* (2004).

*n* = number of patients who completed the assessment.

Taken in aggregate, the scores provide a reasonably detailed and complete characterization of each patient’s language skills. Some of our patients had missing scores in some of those 22 dimensions, either because they could not complete those tasks at all, or because of other practical constraints. Here, we make no assumptions about the reasons why those data are missing, and each task analysis includes only that subset of the patient population who did complete the relevant language task assessment (see Table 2 for the sample sizes in each case). This approach allows us to maximize the sample size in every task analysis.

### MRI data acquisition

Imaging data were collected using either a Siemens 1.5 T Sonata scanner, or a Siemens 3 T Trio scanner. In each case a T_1_-weighted 3D modified driven equilibrium Fourier transform sequence (Deichmann *et al.*, 2004) was used to acquire 176 contiguous sagittal slices with an image matrix of 256 × 224 yielding a final resolution of 1 mm^3^: repetition time/echo time/inversion time = 12.24/3.56/530 ms and 7.92/2.48/910 ms at 1.5 T and 3 T, respectively.

### Data preprocessing

Preprocessed with Statistical Parametric Mapping software (SPM, 2005), these images were spatially normalized into Montreal Neurological Institute (MNI) space using a unified segmentation algorithm (Ashburner and Friston, 2005) optimized for use in patients with focal brain lesions (Seghier *et al.*, 2008), resulting in a binary lesion image for each patient, in standard space. The processing pipeline is described in detail by Seghier *et al.* (2008).

We encoded these lesion images by lesion load (percentage of damage) in a series of anatomically defined regions of the brain: 0% if the region is completely preserved by a patient’s lesion(s), rising to 100% when the region is completely destroyed. We extracted 199 regions for this process, from the Anatomy Toolbox (Eickhoff *et al.*, 2005), the Automatic Anatomical Labelling toolbox (Tzourio-Mazoyer *et al.*, 2002), the ICBM-DTI-81 white-matter labels atlas (Oishi et al., 2011) and the JHU white-matter tractography atlas (Hua *et al.*, 2008). The aim here was simply to cover the whole brain (i.e. grey and white matter in the left hemisphere) in as flexible as possible a manner, so that patients’ lesions could be encoded with minimal *a priori* assumptions concerning what parts of their lesions drive deficits in particular language skills. This conversion also helps to reduce the dimensionality of the lesion data from tens of thousands of voxels to <200 regions. Added to these, were four predictors for: (i) total lesion volume in the left hemisphere; (ii) time post-stroke; (iii) sex; and (iv) age—a total of 203 predictors per patient.

### Model selection

Our goal in this work was to identify and explore any characteristic differences between the monolingual and bilingual groups at the level of brain-behaviour associations. To do this, we first needed to find models that could relate the patients’ demographic and lesion data to their language skills. We restricted our search to linear models, which reduced the model selection process to a feature selection process—finding the right subset of the (203) available predictors to predict each of our 22 language scores. Good models here are models that generate accurate predictions, or in other words, which minimize prediction error. Predictions were made by first training a model with some patient data, using multivariate linear regression to assign coefficients to the model’s predictors, before inverting the model to predict language scores for new patients. We generated predictions for the monolingual patients using leave-one-out cross-validation, or holding one patient’s data out of the data set, training with the rest, then predicting the language score for the held-out patient, and repeating that process once for every single monolingual patient. We generated predictions for the bilingual patients by training with all of the monolingual patient data, and predicting language scores for all the bilingual patients in a single pass.

We selected the best models (or best feature sets) using monolingual data only, with an iterated combination of forward selection and backwards elimination. Starting with a single feature or predictor —time post-stroke—the forward selection phase proceeded by adding, in sequence, the new predictor whose addition most reduced prediction error across the monolingual patient group as a whole. We continued adding predictors until no further improvement was observed. During the backwards elimination phase, we removed the predictor whose removal most reduced model prediction error in monolingual patients, again until no further improvement was observed. We repeated the entire two-phase process until there was convergence on a single feature set, where neither the addition of any new predictor, nor the removal of any current predictor, could reduce prediction error any further. And we repeated the whole process once for every one of the 22 target variables, to find the best predictive models for every language score in monolingual patients. Implemented in Matlab script (2012*a*), this process took ∼48 h on a desktop PC, running Windows 7.

Finally, to ensure that our results were not artefacts of the particular subsets of the 203 available features that we chose when trying to find the best models, we repeated the same analysis with 10 000 models composed of randomly selected features. Specifically, we (i) chose a random group of features (lesion load in some specified set of brain regions, and/or other demographic data); (ii) derived predictions for the monolingual patients from these features using cross-validation; and (iii) derived predictions for the bilingual patients after training with monolingual data only. We repeated the whole process once for each of the 22 language scores, with each of 10 000 random models, for a total of 220 000 tests. This allowed us to check whether any distinctions made between the groups by our best models were also typical of the behaviour of the model-space more generally.

### Characterizing group differences

In Analyses 1 and 2, we characterize group differences by comparing errors on the predictions made for patients in both groups for each of the 22 language scores. The aim here is to establish how predictive lesion-deficit trends learned from the monolingual group might be when predicting prognoses for the bilingual group. In the monolingual patients, predictions are generated by leave-one-out cross-validation, while the bilingual patients’ predictions are made after training with monolingual data only. A focus on prediction errors rather than scores *per se* is appropriate because we expect the scores to depend on the type and severity of the lesions patients have suffered, and also on non-lesion factors such as time post-stroke. Our predictive models naturally take these factors into account. We control for multiple comparisons here using permutation thresholding: repeating the key tests *n* times (*n* = 1000) after shuffling the key variable of interest (the membership of the monolingual or bilingual groups). The effect is to simulate what might be the null distribution of the test statistic. Credible effects should be ‘extreme’ relative to that null distribution.

In Analysis 3, we compare the lesion-deficit associations embodied by the two patient groups. The aim here is to explain the group differences observed in Analyses 1 and 2, either as a result of (i) a simple main effect of group, which does not relate to differences in lesion-deficit associations in the brain; or (ii) enhanced loading in the bilingual group in regions that are also relevant to the monolingual group; or (iii) some sensitivity to damage in the bilingual group in regions that have no prognostic relevance for the monolingual group.

We make most group comparisons with *t*-tests—for independent samples when comparing (i) demographic variables; and (ii) prediction errors across the monolingual and bilingual groups; and for paired samples when comparing native to non-native language scores (within subject) in the bilinguals, and the lesion-deficit correlations (within region) across patient groups. The exception is the comparison of levels of education (years of education beyond the age of 16), where we used the Wilcoxon rank sum test instead. In the other between group comparisons, we assumed equal variances after first confirming that there were no significant differences between the distributions being compared (with Levene’s F-test).

## Results

### Lesion and language data

**Figure 1:**
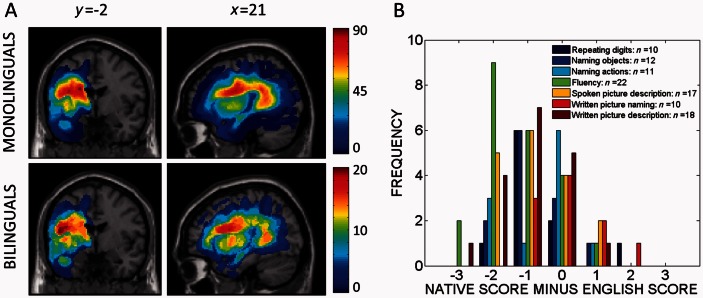
**Patient data**. (**A**) Frequency maps of the two patient groups’ lesions. Two lesion frequency maps in standard (MNI) space, with sagittal and coronal slices centred at *x* = −21 mm, *y* = −2 mm, *z* = 21 mm: the map for the monolingual group is on the *top* and the map for the bilingual group is at the *bottom*. (**B**) Histogram of the differences between the bilingual patients’ language scores in their (non-English) native language and in English. L1 scores were available in 7 of the 22 language assessments considered in the original analyses. The legend indicates both the names of the tasks and the numbers of scores available for comparison in each task. To support comparison across the language tasks, all differences (native language score minus English language score) were standardized to the same range: negative differences indicate that the patient’s language score was better when tested in English than when tested in their own (non-English) native language.

Figure 1A displays the distributions of the lesions suffered by the patients in these two groups; collectively, they cover much of the left hemisphere of the brain, though both groups’ lesions tend to spare most frontal regions. Table 2 reports the scores in each group for all 22 language tasks. Language scores are generally lower in the bilingual group compared to the monolingual group. In addition, for some patients in the bilingual group (Table 3), we collected native language scores for 7 of the 22 language tasks (fluency, spoken and written picture description, repeating digits, naming objects, naming actions and written picture naming, by adapting the relevant parts of Comprehensive Aphasia Test to those non-English languages. In every task but one (written picture naming), the bilingual group appeared to achieve better scores in English than in their native language (Fig. 1B and Table 3).

**Table 3:** Comparing the bilingual patients’ language scores with their non-English native language scores in selected tasks

TASK	*P*	T	Mean L1–L2	*n*
Fluency	<0.001	−4.4	−1.3	22
Repeating digits	0.011	−2.6	−0.7	10
Naming objects	0.018	−2.3	−0.7	12
Naming actions	0.038	−2.1	−0.6	11
Spoken picture description	0.004	−2.9	−0.8	17
Written picture naming	0.907	−0.1	0.01	10
Written picture description	<0.001	−3.5	−1.0	18

Taking just those subsets of patients where native language assessment data were available (with sample sizes reported in the final column), this table compares the native language scores to the English language scores using *t*-tests for paired samples. Histograms for the differences between these scores are depicted in Fig. 3. Negative differences indicate where the patients’ English language scores were better than their (non-English) native language scores on the same task.

L1 = non-English native language score; L2 = non-native English language score; *n* = number of patients for whom both scores were available.

### Analysis 1: Predicting non-native English language scores from native English data

#### Best models

**Figure 2:**
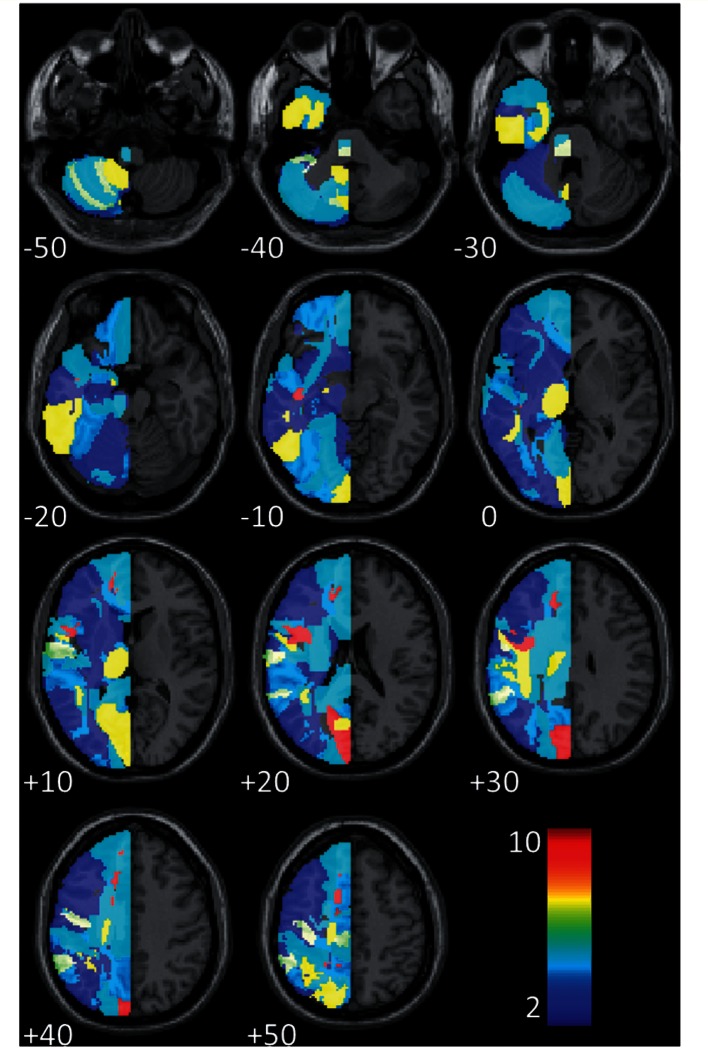
**Frequency of the regions implicated by our best prognostic models for all (22) language tasks**. Because our patient population was restricted to those with left hemisphere stroke only, we only considered regions in the left hemisphere of the brain.

For every dimension of language that we considered, our best predictive models included (i) time post-stroke; and (ii) lesion load in some combination of brain regions. Age at stroke was also included in 5/22 tasks. Figure 2 presents a frequency image of the regions implicated in these (22) predictive models; the smallest model contained 18 predictors, and the largest contained 36. These models can all predict their particular language score with reasonable accuracy in the monolingual group (Table 3), though their performance naturally varies from task to task. And the models also perform well when predicting the scores in the bilingual group, with a higher correlation, albeit often only slightly, between predicted and actual scores in the bilingual group than the monolingual group in 18/22 tasks. The models distinguish ‘better’ from ‘worse’ language outcomes equally well within both patient groups.

**Figure 3:**
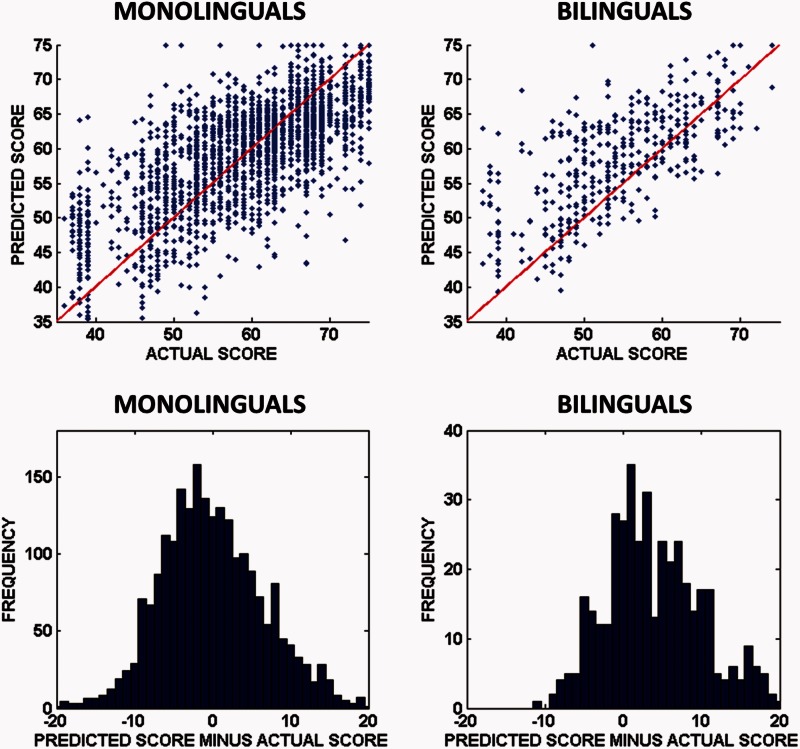
**Predictions and prediction errors, by patient group, in tasks where significant group differences were observed**. *Top*: Scatter plots of the predicted versus actual scores in each of the 13(/22) tasks where significant differences were observed in Table 3; predicted and actual scores are equal along the red line in each case (i.e. perfect predictions would fall along this line). Note that the predictions for the bilingual group (*top right*) tend to fall above the red line, which means that predicted scores tend to be higher than actual scores in these tasks. *Bottom*: Histograms of the prediction errors for predictions made in each of the same 13 tasks; the distribution for the monolingual group is centred close to zero (mean = −0.018), whereas the distribution for the bilingual group is positive (mean = 4.07).

However, the models trained on L1 data often fail in one critical respect: in every task, these models tend to predict higher scores than they should for bilingual patients on average, and this is significant at the 5% level (corrected for multiple comparisons) in 13/22 tasks (Table 4). Focusing solely on those 13 tasks, Fig. 3 illustrates the difference with scatter plots of predicted versus actual scores in each task, and histograms of prediction errors made for all these tasks.

**Table 4:** Best model predictive performance in the monolingual and bilingual patient groups

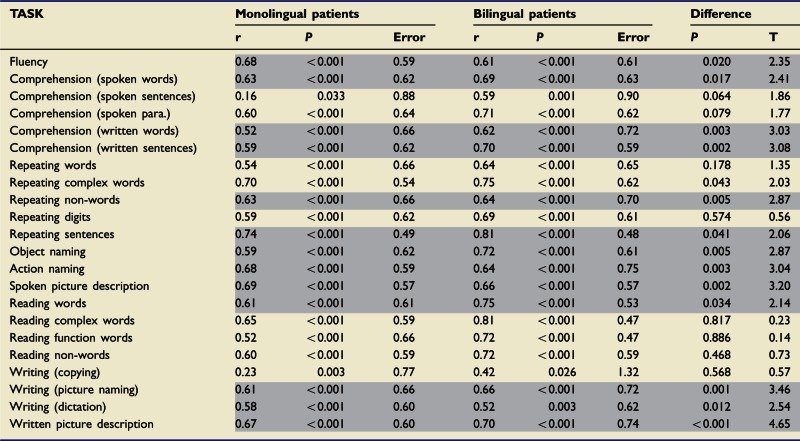

Monolingual performance data are calculated via leave-one-out cross-validation. Bilingual performance data are calculated by predicting bilingual data after training with monolingual data only. Both types of prediction are characterized by (i) correlating predicted scores versus actual scores; and (ii) calculating the mean absolute prediction error for each group. Differences between the prediction error distributions for the monolingual and bilingual groups are characterized by *t*-tests (for independent samples): positive *t*-values here indicate that the bilingual patients’ prediction error distribution is positively shifted relative to the monolingual patients’ prediction error distribution. Highlighted rows indicate where the shift is significant, after correction for multiple comparisons (5% significance level after permutation thresholding: *P* = 0.042).

### Analysis 2: Predicting non-native English language scores from native English data

#### 10 000 Random models

The differences found so far are observed in 13/22 language tasks, when we use our best models to predict those language scores. But are the differences reported in Analysis 1 specific to the particular models, and the particular feature sets, that we used? To check this, we repeated the previous analysis on a further 10 000 models, generated by selecting predictors at random. After conducting the same training and testing procedures as used in Analysis 1, we then observed what distinctions, if any, these random models made between the two patient groups.

Our random models naturally have very variable quality because no effort was made to ensure that the features they used were really predictive of the language scores, but the model space is also consistent with the behaviour we observed in Analysis 1. Specifically, for the 13/22 language tasks where our best models made predictions, which were significantly shifted at the 5% level for the bilingual group (Table 4), we see the same shifts, at the same significance level, in 80.2% of the random models (104 300/130 000). There were no tests at all where we observed a significant group difference in the other direction—i.e. where prediction errors for the bilinguals were negatively shifted relative to those for monolinguals. In other words, most randomly selected models make the same distinction between the patient groups as our best models: those group differences are therefore very unlikely to be an artefact of model selection.

### Analysis 3: Searching for differences at the level of lesion-deficit associations

Here, we searched for group differences at the level of lesion-deficit associations in the brain, which might help to explain the behavioural differences observed so far. Specifically, we searched for differences in the associations between language scores and lesion load in single anatomically defined regions of the brain, and classified those differences according to whether they support either the neural convergence account or an alternative neural divergence account.

The neural convergence account proposes that patients in our L1 and bilingual group share essentially the same language networks but allows a differential loading on different parts of that network. Under this account, the scores of bilingual patients might be worse than expected because of premorbid differences in language proficiency or because their performance is more sensitive to lesion damage in regions which play a similar role for both groups (i.e. where damage causes the same types of language impairment). Neural divergence accounts predict that bilingual language may recruit distinct regions that are not typically implicated in monolinguals’ language networks; on this view, the bilingual group could be worse than expected because they have damage in regions that do not affect language scores in the monolingual group.

To distinguish these two accounts, we used the test proposed by Wetzels and Wagenmakers (2012). In contrast to frequentist tests that only measure evidence for the presence of a correlation, this default Bayesian null hypothesis test quantifies the evidence both in favour of and against the presence of a correlation between two variables. Given only the correlation coefficient and the number of observations made, the test calculates a Bayes Factor, which compares two regression models: the ‘alternative model’, which includes our predictor of interest (i.e. lesion load), and a ‘null’ model which includes only a constant term and a Gaussian noise term. Using the convention proposed by Jeffreys (1961), we interpret Bayes Factors >3 and >10 as indicating ‘substantial’ and ‘strong’ support, respectively for the alternative model (i.e. indicating that the data make the presence of a correlation either 3 or 10 times more likely than its absence). By contrast, Bayes Factors less than one-third or less than one-tenth indicate ‘substantial’ and ‘strong’ support, respectively for the null hypothesis (i.e. that the null hypothesis is 3 and 10 times more likely than the alternative, given the data).

**Figure 4:**
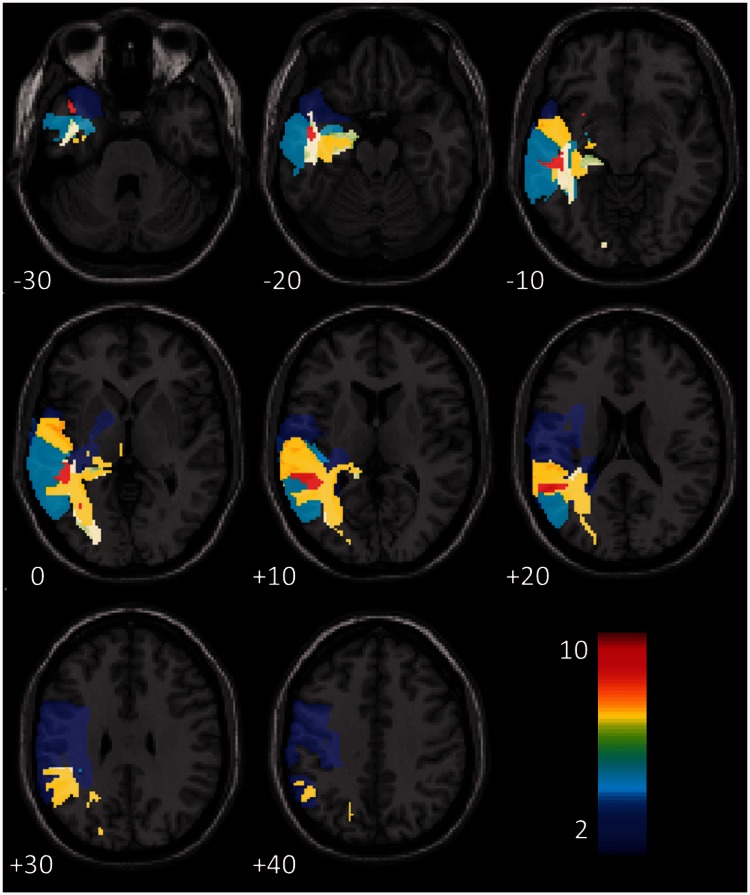
**Frequency map of regions where both patient groups have strong associations between lesion load and one of the 13 critical language tasks**. The frequency of each region (max = 11) refers to the number of tasks (/13) where both patient groups displayed strong evidence of a correlation between task score and lesion load in that region.

Across all of the 13 language tasks considered here (those where significant group differences were observed in Analysis 1), there are 129 cases where there is ‘strong’ evidence for a correlation (Bayes Factor >10), in the bilingual group, between some language score and lesion load in some specific region of the brain. In 127/129 cases, we find the same association (Bayes Factor >10) in the monolingual group. In these regions, increasing damage is associated with increasingly severe deficits in the same language skills in both patient groups (Fig. 4). Critically, the coefficients of the associations in these regions are significantly more negative for the bilingual group than they are for the monolingual group (df = 128, t = 16.24, *P* < 0.001). In other words, the differences observed in Analysis 1 plausibly reflect enhanced sensitivity to lesion damage in the bilingual group in regions that play a similar role in the monolingual group.

In the two cases where the correlation for the bilingual group was strongly supported, but the correlation for the monolingual group was not strongly supported, the Bayes Factors for the monolingual group were 8.55 and 0.96, respectively. In the first case, there is still ‘substantial’ (Jeffreys, 1961) evidence in favour of a correlation, and in the second, there is no solid evidence either way. In sum, across all of the 13 language tasks that we identified in Analysis 1, we found no single region where scores for the bilingual group are sensitive to damage and scores for the monolingual group are not. This finding is not predicted by neural divergence accounts but is consistent with the neural convergence account of bilingual language in the brain.

## Discussion

Across a large range of language tasks, from reading single words to repeating full sentences, our bilingual patients appear to have lower English language scores than predicted from lesion-behaviour data in the monolingual group. These differences might be explained in at least three ways.

The first explanation is that the bilingual patients appear worse than expected because they are assessed in their non-native language, whereas the monolingual patients are assessed in their native language. If this is true, we would expect the bilingual patients to get better scores when tested in their non-English native languages. What data we have here suggests the reverse: that the bilinguals may actually have been better at English than in their native languages (Table 3). There may be testing confounds at play here: though our tests were a subset of the Comprehensive Aphasia Test, the actual exemplars used for naming may not have been typical of the referents of target nouns in the patients’ native languages. Even if they were typical, those referents may have been less common in the patients’ native languages than in English (e.g. a Spanish patient might find it harder to name a ‘hedge’ in Spanish than in English, because hedges are a more common feature of Britain than of Spain). On the other hand, our bilingual patients were immersed in an English-speaking environment and typically used English most of the time premorbidly (Table 1), so the results in Table 3 may reflect poorer native language function premorbidly rather than greater post-morbid impairment for that language. If so our data are in line with the typical, parallel pattern of language recovery post-stroke (Paradis, 2004). Post-stroke differences in language use may also contribute to the observed data (Table 1), and as residents of the UK, what speech and language therapy they had would likely have emphasized English. In other words, though our data could be confounded, they are also potentially plausible, and they discourage the interpretation of the group differences we see here as an artefact of testing in the non-native language.

One alternative explanation for those differences is that they occur because the bilingual patients had lower levels of premorbid language proficiency (in any language) than the monolingual patients. In the neurologically normal population, bilingual speakers typically recall verbal materials more poorly (Fernandes *et al.*, 2007; Bialystok *et al.*, 2009) and recognize and produce words more slowly (Portocarrero *et al.*, 2007; Bialystok *et al.*, 2008), than monolingual speakers, even when they use their native language (Ivanova and Costa, 2008). As mentioned previously, differential practice and experience may be one source of such differences, as bilingual speakers use each of their languages proportionately less of the time than monolingual speakers of those languages. We had no objective measures of premorbid proficiency for any of the patients, but premorbid proficiency differences could plausibly account for the post-stroke differences we observed in Analysis 1.

However, this explanation does not predict the differences we observed at the level of lesion-symptom associations in Analysis 3. A main effect of group membership (bilingual versus monolingual) need not imply any differences at all in terms of the association between increasing damage in key brain regions, and increasing symptom severity. This result is consistent with the claim that monolingual and bilingual brains share essentially the same language networks, though bilingual brains may exhibit enhanced loading and so increased sensitivity to damage in some parts of that network—as proposed in the neural convergence account of the bilingual brain (Green, 2003; Consonni *et al.*, 2013). By contrast, we could find no instances where there was ‘strong or better’ support (Jeffreys, 1961) for neural divergence accounts, which predict that the bilingual brain should recruit different regions for processing a non-native, second language (including the procedural/declarative model, which predicts that such differences will emerge principally in sentence processing tasks). We suggest that the most plausible account of the group differences we see here is that they flow from some combination of (i) premorbid proficiency differences; and (ii) enhanced loading (and thus enhanced sensitivity to damage) in a common network of regions recruited to implement language in both patient groups.

We note several potential limitations of our results, some relating to our sample, and others concerning out analytic method. First, although we sought to control these analyses as carefully as possible, there remain potential confounds that we cannot address with the current patient population and that could be usefully explored in future work. As a general point, our sample provides very little data on the patients’ experience of speech and language therapy. We note, however, that in future work, when the patient sample is partitioned into those that have experienced a given therapy and those that have not, prognostic models based on the latter provide a way to assess the effectiveness of the therapeutic intervention. Our current bilingual group also mixes bilinguals of different language histories, with a mixture of simultaneous and sequential bilinguals (a distinction that could be pertinent to language recovery) (Hull and Vaid, 2007), although none of the bilingual patients learned English from birth. In fact, we found no evidence that language history mediates the group differences observed here: there were no significant associations between any of the language history variables we collected (as listed in Table 1), and prediction errors in any of the 13 tasks where we saw significant group differences. But our language history data were incomplete for many of the bilingual patients, and the sample itself is neither particularly large, nor selected to drive a balanced, within-group comparison at this level. Therefore we cannot dismiss the relevance of language history here with any real confidence.

One more methodological limitation stems from the imbalance in the sizes of the two patient groups (174 monolinguals versus 33 bilinguals). We had little control over this demographic distribution and used what data we had. But the split is certainly inefficient, because the power of between-groups comparisons is maximized when both groups are the same size, though the current design is still more powerful than a balanced comparison between two groups of size 33. Nevertheless, the comparatively small size of our bilingual sample encourages caution when interpreting these results, unless and until they can be replicated in larger patient populations.

Another potential limitation stems from the model-dependent nature of Analyses 1 and 2, where we use model prediction errors to characterize group differences. This approach is useful because our models naturally take many of the relevant differences between these patients into account, and the results of Analysis 2, where we measured the behaviour of many random models, suggest that our best models’ behaviour is typical of most alternative models we might have chosen. But whenever we use this approach, there is the risk that newer, better models will be found that change our substantive conclusions. This risk is ever-present in cross-sectional group studies with stroke patients, because no two patients are the same and no two patients ever suffer identical stroke damage, but its likely significance should diminish as our sample grows larger.

Normal bilingual speakers tend to show a lower level of language proficiency compared to monolingual speakers and our data on bilingual stroke patients conform to this pattern. But weaker language skills in the normal brain may nonetheless be associated with enhanced cognitive control skills. Normal bilingual speakers, for example, are poorer at processing sentences in noise relative to monolingual speakers (Shi, 2010) but are better able to resist the effects of a concurrent distracting and irrelevant auditory sentence when there is a reliable cue to selection of the correct target (Filippi *et al.*, 2012). The life-long exercise of controlling weaker linguistic representations and avoiding interference between two languages may underlie advantages reported for older bilingual speakers in tasks involving cognitive control (Bialystok *et al.*, 2009). Such advantages may be an important mediator of the neuroprotective effects of bilingualism in the ageing brain and enhance cognitive reserve (Stern, 2009) as intimated by studies reporting a delay in the onset of symptoms of Alzheimer’s disease (Bialystok *et al.*, 2007; Chertkow *et al.*, 2010; Craik *et al.*, 2010; Alladi *et al.*, 2013) and mild cognitive impairment (Bialystok *et al.*, 2014). Whether a similar advantage obtains following stroke is an open and interesting empirical question. Our data suggest that if there is any such advantage it seems not to reduce the impact of lesion load in bilingual stroke patients.

However, given the need to control interference between two languages, damage to the networks involved in the cognitive control of language may limit the extent of bilingual language recovery. In a single case study using dynamic causal modelling of neuroimaging data (Abutalebi *et al.*, 2009), improvements in picture naming in the non-native language following treatment in that language were shown to be mediated by increased connectivity of regions involved in language control (e.g. left inferior frontal regions, anterior cingulate cortex and left head of caudate) (Abutalebi and Green, 2007), and those involved in picture naming (inferior frontal gyrus and fusiform gyrus) (Demonet *et al.*, 2005). By contrast, no increase in such connectivity was observed for the untreated native language that did not improve. If substantiated by further research, this result suggests that indices of the integrity of white matter tracts subserving language control may contribute to improved predictions of language recovery in bilingual stroke patients more generally.

In conclusion, the current results, both the differences that we see in the groups’ language scores and the associated differences at the level of lesion-deficit associations in the brain, appear robust in our data. Our models tend to be over-optimistic about the bilingual group as a whole, but they distinguish ‘better outcomes’ from ‘worse outcomes’ equally well within both patient groups. If these results are robust to validation in larger samples of patients, the implication is very encouraging clinically: given a potentially simple correction, prognostic models built for monolingual stroke patients can be generalized to bilingual stroke patients.
