# Corrigendum

**DOI:** 10.1093/brain/awx079

**Published:** 2017-07-26

Thomas M. H. Hope, ‘Ōiwi Parker Jones, Alice Grogan, Jenny Crinion, Johanna Rae, Louise Ruffle, Alex P. Leff, Mohamed L. Seghier, Cathy J. Price and David W. Green. Comparing language outcomes in monolingual and bilingual stroke patients. Brain 138; 1070–1083; doi:10.1093/brain/awv020

The Funding section in the above manuscript should have read:

This work was funded by the Wellcome Trust, the James S. MacDonnell Foundation, the Medical Research Council, (MR/M023672/1/Medical Research Council/United Kingdom), and the Stroke Association (165018).

This has now been corrected online, and the authors apologize for the error.

